# Design and Modeling of a Test Bench for Dual-Motor Electric Drive Tracked Vehicles Based on a Dynamic Load Emulation Method

**DOI:** 10.3390/s18071993

**Published:** 2018-06-21

**Authors:** Zhe Wang, Haoliang Lv, Xiaojun Zhou, Zhaomeng Chen, Yong Yang

**Affiliations:** 1 State Key Laboratory of Fluid Power and Mechatronic Systems, Zhejiang University, Hangzhou 310027, China; wzhe@zju.edu.cn (Z.W.); lhl801@zju.edu.cn (H.L.); chernzm@zju.edu.cn (Z.C.); 2 Beijing Institute of Astronautical Systems Engineering, Beijing 100076, China; yyongpaper@gmail.com

**Keywords:** dual-motor electric drive tracked vehicle, dynamic load emulation, speed tracking control method, test bench model

## Abstract

Dual-motor Electric Drive Tracked Vehicles (DDTVs) have attracted increasing attention due to their high transmission efficiency and economical fuel consumption. A test bench for the development and validation of new DDTV technologies is necessary and urgent. How to load the vehicle on a DDTV test bench exactly the same as on a real road is a crucial issue when designing the bench. This paper proposes a novel dynamic load emulation method to address this problem. The method adopts dual dynamometers to simulate both the road load and the inertia load that are imposed on the dual independent drive systems. The vehicle’s total inertia equivalent to the drive wheels is calculated with separate consideration of vehicle body, tracks and road wheels to obtain a more accurate inertia load. A speed tracking control strategy with feedforward compensation is implemented to control the dual dynamometers, so as to make the real-time dynamic load emulation possible. Additionally, a MATLAB/Simulink model of the test bench is built based on a dynamics analysis of the platform. Experiments are finally carried out on this test bench under different test conditions. The outcomes show that the proposed load emulation method is effective, and has good robustness and adaptability to complex driving conditions. Besides, the accuracy of the established test bench model is also demonstrated by comparing the results obtained from the simulation model and experiments.

## 1. Introduction

Tracked vehicles, with their advantages of perfect dynamic performance under complicated driving conditions, are widely used in agriculture, construction, the military and many other fields. Compared with traditional tracked vehicles, dual-motor electric drive tracked vehicles (DDTVs) have been attracting increasing attention due to their high transmission efficiency, economical fuel consumption, silent driving performance and easy maintenance. 

Currently, many advanced control technologies related to hybrid powertrain system have been successfully applied to passenger cars, while the applications for tracked vehicles are relatively narrow and immature. In this case, scholars have undertaken a large number of related studies, where they most concentrate on the drive control strategy (DCS) of the dual-motor under different driving conditions [1,2,3,4], and the energy management strategy (EMS) for power distribution such as the rule-based control strategy [5] and the optimization-based control strategy [6,7,8,9]. However, these strategies must be validated by other methods before they are accepted and equipped on real vehicles. Verification is an inevitable process to provide a reliable scientific basis for the development of vehicle technologies or products. Currently, the validation of DCS is mostly conducted by real road tests [2,10], and the verification of EMS is mainly based on software simulations [7,8]. 

There are mainly three ways for verification, namely system simulations based on software such as ADVISOR, CRUISE and RECURDYN [11,12,13], bench tests and real road tests [10,14]. Software simulation has the advantages of low cost, high efficiency and flexibility, but the complex structure of tracked vehicles makes it difficult to accurately mathematically model or describe them. Usually, an extra validation procedure is needed after the software simulation. As for real road tests, they incur in high costs to build the specified test ground and have a limited range of testing conditions though they have perfect fidelity. Bench tests, a combination of the above two ways, are regarded as a flexible and efficient approach that enables researchers to conduct a large bandwidth experiments just by simply reconfiguring the bench parameters corresponding to different test purposes. In this way, it is possible for researchers to study and evaluate new technologies in academic environments [15].

Bench tests can not only shorten the development cycle of new vehicle technologies but also reduce the test cost. However, the design of a test bench itself can be very complicated. At present, there are many references focusing on the construction of test benches for hybrid electric passenger cars [16,17,18,19,20]. As for tracked vehicles, the existing literature concerned with test benches is still scarce, and the few accessible investigations mainly concentrate on traditional tracked vehicles [21,22]. In this case, the development of new technologies for DDTVs may be affected. Hence, a test bench for DDTVs is both necessary and urgent.

In terms of DDTV test benches, emulation of the dynamic load may be the most critical part. A test bench should have the ability of emulating the loads the same as those when the vehicle runs on a real road to guarantee that real-time constraints can be matched. On the condition that the load emulated on a test bench is similar to a real-road one within a certain accuracy range, results obtained from the test bench could be reasonable and acceptable.

When a DDTV runs on a real road, the main load is composed of two parts. One is the road load, which is caused by the whole resistive forces opposing the vehicle’s motion, consisting of rolling friction resistance, aerodynamic drag and grading resistance. The other one is the inertia load caused by the vehicle’s mass, and emerges in the acceleration or deceleration driving conditions. A DDTV test bench should be capable of emulating both the road load and inertia load. Drum test benches used to be widely applied in the performance investigation of wheeled vehicles [23]. However, with the development of electric and control techniques, test benches increasingly use dynamometers to achieve better load emulation performance. Dynamometers perform well in static load tests of machines, but for testing variable speed drives, static load tests are not sufficient and dynamic load tests are required [24,25]. Usually, on a drive-line test bench, the dynamometer is utilized to emulate the road load, and the flywheel disks provide adjustable inertias and are used for emulating the inertia load [26,27]. Nevertheless, researchers have found that flywheel boxes with large inertia, high working speed and high dynamic balance requirement, are hard to manufacture and have safety risks [13]. In addition, different tested vehicles have different inertias, resulting in the reconfiguration of the flywheel disks to match the desired one. The fact that the inertias of flywheel disks are in discrete sizes will make the reconfiguration troublesome [28,29]. In this case, the flywheel box is usually eliminated, and instead, the dynamometer is controlled to emulate both the inertia load and the road load simultaneously. 

There are several approaches to controlling dynamometers for dynamic load emulation. The inverse mechanical-dynamics (IMD) principle used to be widely applied due to its easy implementation [24,30]. However, in view of the sampling effects of digital microprocessors, the output of the inverse model will be noisy due to the existence of the derivative terms in the inverse transfer function. An approach with compensator based on system transient response is introduced in reference [31]. This compensator can be fully designed by applying parametric system identification to experimental data from transient system step tests. This method shows good performance for fast dynamics emulation at high speeds. References [32,33,34] have conducted adequate research on a nonlinear approach with the PI estimator, this method makes it more robust towards impacts of parameter variations and unmolded dynamics. It is designed to investigate unknown friction dynamics in the test bench and to compensate nonlinear friction. This paper presents a speed tracking algorithm with feedforward compensation. It applies a feedback controller together with a feedforward compensator for the calculation of demand load torque of the dynamometer [35,36]. The method can preserve the real road load model dynamics when used in a closed-loop system. 

The aim of this work is to investigate the creative application of this dynamic load emulation method on the design of a DDTV test bench featuring high power and large inertia. It will be a reference for potential researchers in related field. Additionally, a MATLAB/Simulink model of the DDTV test bench is established based on detailed derivation of the system dynamics.

The organization of this paper is as follows. In Section 2, the structure of a series DDTV is introduced. In Section 3, road resistance model of a DDTV is built and the vehicle’s total equivalent rotational inertia calculated at the drive wheel is performed. Platform design, dynamic load emulation method, control strategy and test bench system modeling are illustrated in Section 4. Section 5 introduces the experiment system, including general setup of the bench, important device parameters and different test contents. Section 6 shows the experimental results under different test conditions for the effectiveness validation of the proposed method and the accuracy verification of the established test bench model. Finally, conclusions are given in Section 7.

## 2. Structure of a DDTV

**Figure 1:**
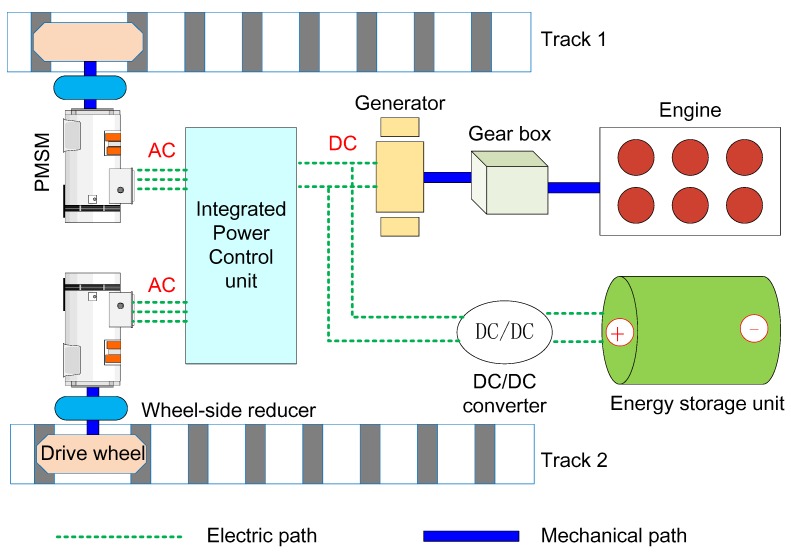
Schematic diagram of a DDTV structure.

The hybrid powertrain system is usually divided into three types according to their different structures. They are series systems, parallel systems and serial-parallel systems [37]. This paper focuses on a series DDTV and its schematic structure is shown in Figure 1. Two tracks are attached to corresponding drive wheels. The dual drive wheels are separately driven by two permanent magnet synchronous motors (PMSM). Electric energy of these two motors is provided by the engine-generator set (EGS) and the energy storage unit (battery pack or supercapacitor). In this case, the vehicle’s dynamic performance features like acceleration, steering and braking could be achieved by independently controlling the dual side motors. The integrated power control unit is used for power allocation between the EGS and energy storage unit, thus ensuring the superior fuel economy.

## 3. Analysis of DDTV Dynamics

### 3.1. Longitudinal Dynamics Analysis

There are three kinds of forces acting on a DDTV when running on a real road. They are the driving force provided by drive motors, braking force offered by a mechanical brake device or drive motor (in regenerative braking), and road resistance. The longitudinal dynamics equation can be denoted as:

(1) $$F_{d}-F_{R}=m\frac{dv}{dt}$$

(2) $$\left\{ \begin{array}{l} F_{R}=F_{f}+F_{s}+F_{w}\\ F_{f}=mg\cos\theta \cdot f\\ F_{s}=mg\sin\theta \\ F_{w}=\frac{1}{2}\rho C_{D}Av^{2} \end{array}\right.$$

where *F_d_*, *F_R_* refer to the driving force and road resistance, respectively, N. *F_f_*, *F_s_*, *F_w_* are the rolling friction resistance, grading resistance and aerodynamic drag, N. *m* is the vehicle mass, kg. *f* is the rolling friction resistance coefficient. *g* is the gravity, m/s^2^, *θ* is the slope angle, rad. ρ is the air density, Ns^2^/m^4^. *C_D_* is the coefficient of aerodynamic drag, *v* is the vehicle running speed, km/h. *A* is the frontal area of the vehicle, m^2^.

For convenient analysis, these forces are multiplied by the radius of drive wheel (*R*), thus converting to the corresponding torques acting on the drive wheels:

(3) $$\bar{T_{d}}-\bar{T_{R}}=\bar{J_{eri}^{total}}\frac{d\omega_{r}}{dt}$$

where $\bar{T_{d}}$, $\bar{T_{R}}$ denotes the drive torque and road resistance torque, respectively, Nm. $\bar{T_{i}}=\bar{J_{eri}^{total}}\frac{d\omega_{r}}{dt}$ is inertia torque. $\bar{J_{eri}^{total}}$ is the total equivalent rotational inertia (ERI) of the vehicle calculated at drive wheels, kg·m^2^. $\omega_{r}$ is the rotational speed of drive wheel in rad/s.

Equation (3) indicates that when a DDTV runs on a real road, the main load is composed of two parts. One is the road load caused by the road resistance torque. The other one is the inertia load caused by the vehicle’s mass, and emerges in the acceleration or deceleration driving conditions.

From references [15,38], we know that under the same standard driving cycle, inertia torque shows more intense transient changes than road resistance torque, meanwhile, the inertia torque is usually larger than the road resistance torque when a sudden change in vehicle speed occurs. Given these facts, it is crucial to rigorously take inertia torque into consideration for getting accurate test results while emulating real-road driving conditions on a DDTV test bench.

### 3.2. Calculation of Equivalent Rotational Inertia

**Figure 2:**
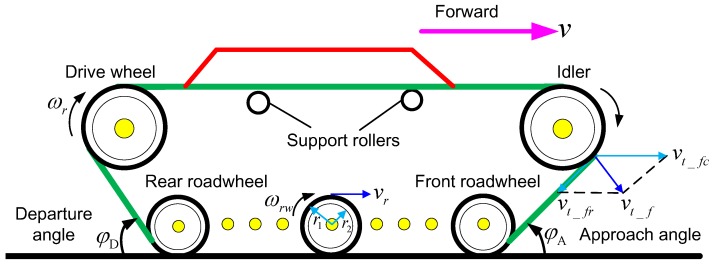
Schematic diagram of forces acting on track and wheels.

According to Equation (3), the inertia torque has a close relationship with the total ERI, so when calculating the inertia torque, a more accurate value of the total ERI is required. A DDTV mainly consists of three components including the body, tracks and wheels, as shown in Figure 2. When a DDTV runs on a real road, the total kinetic energy is made up of the translational kinetic energy stored in the moving body and the rotational kinetic energy stored in the wheels and tracks. It is worth noting that the track of a DDTV accounts for a relatively large proportion of vehicle weight. Moreover, the speed of each part is not the same due to its irregular shapes, so its rotational kinetic energy should not be ignored [21]. In this case, the equivalent rotational inertia of the body, tracks and wheels calculated at the drive wheel are implemented separately in this work based on the kinetic energy conservation theory.

(1) The ERI of Vehicle Body

According to the energy conservation theory, the relationship between translational kinetic energy and rotational kinetic energy can be expressed as follows:

(4) $$\frac{1}{2}mv_{v}{}^{2}=\frac{1}{2}J_{eri}^{b}\omega_{r}{}^{2},\;v_{v}=\omega_{r}R$$

Subsequently, the ERI of the vehicle body calculated at the drive wheel can be obtained:

(5) $$J_{eri}^{b}=mR^{2}$$

where, *R* is the radius of drive wheel, m. *m* is the mass of vehicle body, *V_v_* is the speed of body, m/s, the same as the vehicle running speed *V*. $J_{eri}^{b}$ represents the ERI of the vehicle body calculated at the drive wheel.

(2) The ERI of Track

A track is divided into four parts for individual analysis of the ERI calculated at the drive wheel because of different dynamic characteristics. They are the upper part, the lower part, the front part, and the rear part. Since the lower part is always attached to the ground, it has no translational kinetic energy as well as rotational kinetic energy, The ERI of this part can be neglected. As for the other three parts, taking the front part as an example to introduce the calculation method. The schematic diagram of forces acting on the front part is shown in Figure 2. The dynamic equations can be described as follows:

(6) $$\left\{ \begin{array}{l} \vec{v_{t\_f}}=\vec{v_{t\_fc}}+\vec{v_{t\_fr}}\\ v_{t\_fc}=v_{t\_fr}=\omega_{r}R\\ \frac{1}{2}m_{t\_f}v_{t\_f}{}^{2}=\frac{1}{2}J_{{}_{eri}}^{t\_f}\omega_{r}{}^{2} \end{array}\right.$$

Subsequently, the ERI of the front part of the track calculated at the drive wheel can be obtained:

(7) $$J_{{}_{eri}}^{t\_f}=2m_{t\_f}R^{2}(1-\cos\varphi_{A})$$

where, $\vec{v_{t\_f}}$, $\vec{v_{t\_fc}}$, $\vec{v_{t\_fr}}$ refer to the absolute speed, pulling speed and relative speed, respectively, m/s. $m_{t\_f}$ is the mass of the front part of the track. $J_{{}_{eri}}^{t\_f}$ represents the ERI of the front part track calculated at the drive wheel. $\varphi_{A}$ is the approach angle of the track.

ERI of the other two parts of the track calculated at the drive wheel is acquired using the same method above. The results are:

(8) $$J_{{}_{eri}}^{t\_u}=4m_{t\_u}R^{2}$$

(9) $$J_{{}_{eri}}^{t\_r}=2m_{t\_r}R^{2}(1-\cos\varphi_{D})$$

where, $m_{t\_u}$, $m_{t\_r}$ are respectively the mass of the upper part track and the rear part track. $J_{{}_{eri}}^{t\_u}$, $J_{{}_{eri}}^{t\_r}$ represent the ERI of the upper part track and the rear part track calculated at the drive wheel, respectively. $\varphi_{D}$ is the departure angle of the track.

Based on the Equations (7)–(9). The ERI of single-side track calculated at drive wheel can be denoted as:

(10) $$J_{{}_{eri}}^{t}=2m_{t\_f}R^{2}(1-\cos\varphi_{A})+2m_{t\_r}R^{2}(1-\cos\varphi_{D})+4m_{t\_u}R^{2}$$

(3) The ERI of Roadwheels

A roadwheel of a tracked vehicle is ring-shaped and its mass is concentrated on the edge of the wheel. The dynamic equations can be described as follows:

(11) $$\left\{ \begin{array}{l} \frac{1}{2}n_{r}J_{r}\omega_{rw}{}^{2}=\frac{1}{2}J_{eri}^{r}\omega_{r}{}^{2}\\ v_{v}=\omega_{r}R=\omega_{rw}r_{1}\\ J_{r}=\frac{1}{2}m_{r}(r_{1}{}^{2}+r_{2}{}^{2}) \end{array}\right.$$

Then, the ERI of roadwheels calculated at the drive wheel can be obtained:

(12) $$J_{eri}^{r}=\frac{1}{2}n_{r}m_{r}(r_{1}{}^{2}+r_{2}{}^{2})\frac{r_{2}{}^{2}}{r_{1}{}^{2}}$$

where, $m_{r}$ is the mass of the roadwheel. $\omega_{rw}$ is the speed of the roadwheel. *r_1_*, *r_2_* are the radius of the outer circle and inner circle, respectively. $J_{r}$ is the rotational inertia of the roadwheel. $n_{r}$ is the number of roadwheels equipped on a single-side track. $J_{eri}^{r}$ represents the ERI of the whole single-side roadwheels calculated at the drive wheel.

According to Equations (5), (10) and (12), the total ERI of a DDTV calculated at the dual-side drive wheels is:

(13) $$\bar{J_{eri}^{total}}=J_{eri}^{b}+2J_{eri}^{t}+2J_{eri}^{r}$$

## 4. Modelling the Test Bench System

### 4.1. Design of the Test Bench

**Figure 3:**
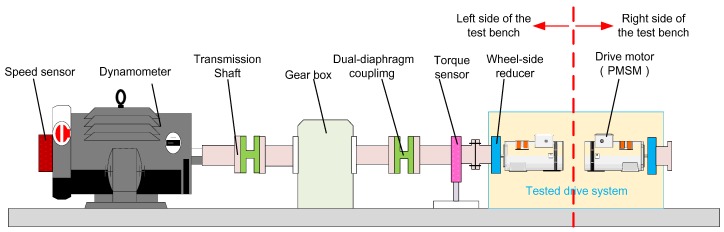
Schematic diagram of the DDTV test bench (The left side is only displayed due to symmetrical arrangement of the test bench).

The schematic diagram of the designed DDTV test bench is shown in Figure 3. Due to symmetrical arrangement of the system, only the left side is displayed in this figure. From the figure, it is clear that the test bench is composed of the vehicle drive system and the load emulation system. The vehicle drive system is the test object. The load emulation system consists of the dynamometer, gearbox, transmission shaft, torque sensors and speed sensors.

Generally, a test bench should have the ability of emulating a large range of dynamic loads because of different type of the tested vehicles or different test conditions. For example, the dynamometer needs higher torque performance to emulate the dynamic load under the driving condition of acceleration or steering than that under the driving condition of uniform speed. If the dynamometer connects directly to the drive system, the rated torque of the dynamometer is always chosen according to the required peak load torque. In this case, it will apparently enhance the expenses of the dynamometer. Hence, a gearbox is added between the dynamometer and the drive system in this work. The gearbox adopts spray oil to lubricate the rolling bearings and meshing gears. It must be pointed out that ratio and inertia of the gearbox are the only two factors considered in this paper. Other gearbox dynamics, such as backlash which will bring about slight nonlinear effect to transmission system [39], is assumed to be negligible. The torque sensor is arranged at the end of the output shaft connected to the vehicle’s drive wheel so that the output drive torque of the vehicle can be measured directly. Additionally, the double-diaphragm coupling is used for linking the shafts because of its superior performance on eliminating the concentricity deviation which inevitably occurs in long shaft transmission. 

### 4.2. Speed Tracking Method

**Figure 4:**
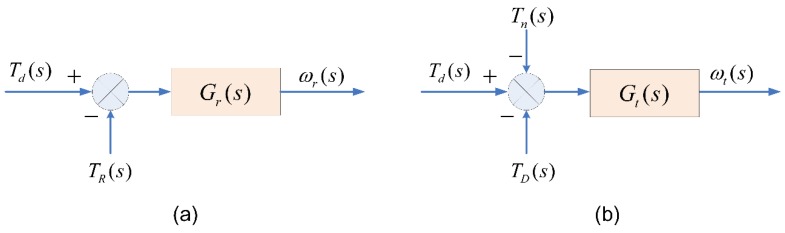
The transfer function model of the vehicle. (**a**) On a real road; (**b**) on a test bench.

When developing a test bench for a DDTV, the design of its control system is important. Accurately emulating the dynamic load of a vehicle when it runs on a real-road on a test bench is the most crucial issue. In this work, both the road load and the inertia load are emulated by dynamometer taking a single side drive wheel as the analysis object:

(1) On one hand, when a DDTV straightly runs on a real road, the transfer function model of the vehicle is shown in Figure 4a, and can be described as:
  (14) $$\frac{\omega_{r}(s)}{T_{d}(s)-T_{R}(s)}=G_{r}(s)$$
  where, $\omega_{r}$ is the speed of drive wheel on a real road in rad/s, here we call it reference speed. $T_{d}$ is the drive torque of a single side drive wheel, $T_{R}$ is the resistance torque acting on a single side drive wheel.
(2) On the other hand, when a DDTV is tested on a bench, the transfer function model of the vehicle is shown in Figure 4b, and can be described as:
  (15) $$\frac{\omega_{t}(s)}{T_{d}(s)-[T_{D}(s)+T_{n}(s)]}=G_{t}(s)$$
  where, $\omega_{t}$ is the actual speed of drive wheel on the test bench in rad/s, here we call it actual speed. $T_{D}$ is the load torque imposed by the dynamometer, $T_{n}$ is the frictional resistance torque of the platform transmission system, mainly consists of bearing viscous friction, sliding friction caused by gear teeth meshing and wind friction.

The primary purpose of load emulation on a test bench is to ensuring that the Equation (14) and the Equation (15) are equivalent. To achieve this goal, inverse mechanical-dynamics (IMD) principle and speed tracking method are usually applied:

(1) IMD Principle

**Figure 5:**
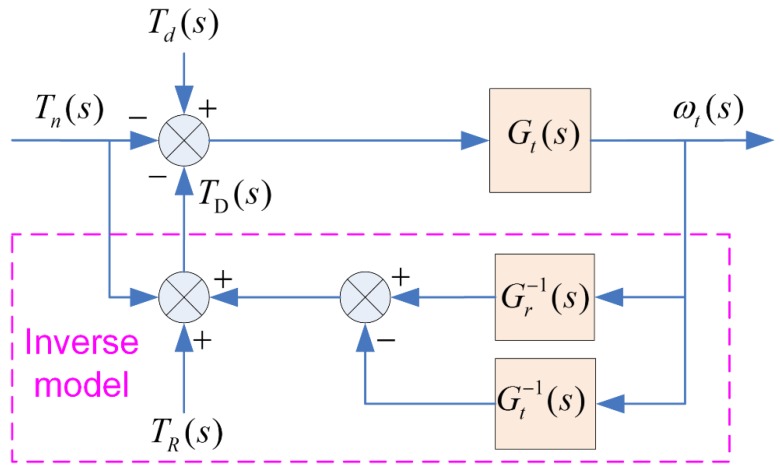
Control schematic diagram of IMD principle.

As for IMD principle, its control schematic diagram is shown in Figure 5, the goal is to ensure the relationship between the actual speed $\omega_{t}$ and the drive toque $T_{d}$ on a test bench results in the dynamics given by Equation (14). Thus, the load torque $T_{D}$ can be easily obtained by Equation (16). This method is simple and can give good results in continuous time. While, in view of the sampling effects of a digital microprocessor, the output of the inverse model will be noisy because of the existence of the derivative terms in the inverse transfer function. Besides, the system would be instable if the desired emulated vehicle inertia was twice larger than the inertia of the test bench itself [40]:

(16) $$T_{D}(s)=\;\omega_{t}(s)(G_{{}_{r}}^{-1}(s)-G_{t}^{-1}(s))+T_{R}(s)-T_{n}(s)$$

**Figure 6:**
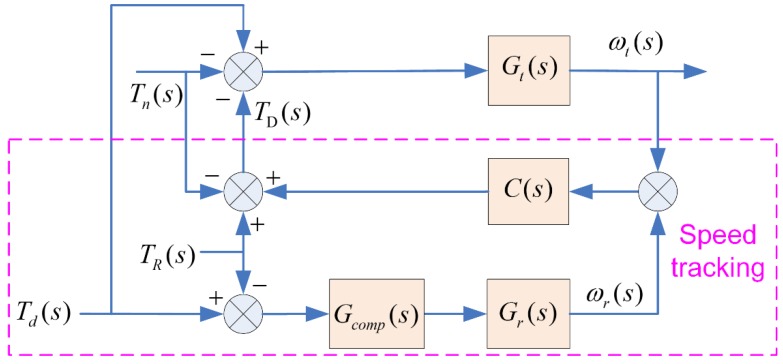
Control schematic diagram of speed tracking method.

Due to the wide range of the desired emulated inertias of different tested DDTVs, the IMD principle method is inaccessible as it only performs well when the desired emulation inertia is two times less than that of the test bench. Therefore, in this work, we apply the speed tracking method for load emulation. Based on the original theory proposed by Hakan et al. [35,41], a speed tracking method with feedforward compensator is presented, the principle of this method is shown in Figure 6. 

(2) Speed Tracking Method

In Figure 6, C(s) and $G_{comp}(s)$ are, respectively, the feedback controller and feedforward compensator. They are used for the speed control of the dynamometer. In this method, the drive torque $T_{d}$ is applied to drive a desired dynamic $G_{r}(s)$ for obtaining the reference speed $\omega_{r}$, and this speed should be followed by the actual rotation speed $\omega_{t}$ of the test bench. By comparing the reference speed $\omega_{r}$ and the actual speed $\omega_{t}$, the feedback controller C(s) outputs the reasonable dynamometer torque $T_{D}$.

According to Figure 6, the dynamic model of the test bench can be denoted as:

(17) $$\frac{\omega_{t}(s)}{T_{d}(s)-[T_{D}(s)+T_{n}(s)]}=G_{r}(s)G_{comp}(s)\frac{C(s)G_{t}(s)}{1+C(s)G_{t}(s)}$$

when the feedforward compensator is designed as $G_{comp}(s)={\left[\frac{C(s)G_{t}(s)}{1+C(s)G_{t}(s)}\right]}^{-1}$, the actual speed $\omega_{t}$ can track the reference speed $\omega_{r}$ in real time. Thus, Equation (14) is equivalent to Equation (17). Remarkably, $G_{comp}(s)$ just contains the parameters of the test bench system itself and has nothing to do with the parameters of the tested vehicle, which makes the compensator constant in spite of different tested specimens. In addition, this method can preserve the pole–zero structure of the desired system dynamics and suitable for discrete-time applications [18,38].

### 4.3. System Modeling

**Figure 7:**
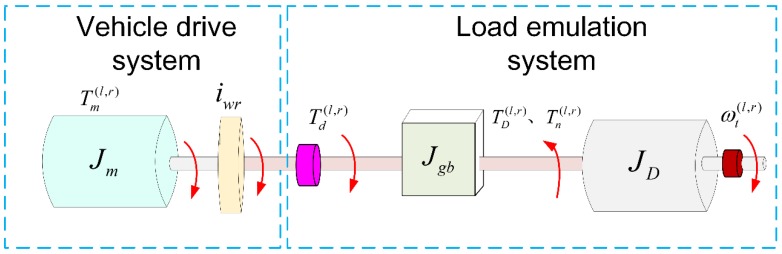
Dynamics of a single side test bench.

According to Section 4.2, the transfer function of the test bench itself should be known when implementing the speed tracking method. Since the components of the left-side test bench are the same as those of the right-side, the single side system is taken as an example for modeling and analysis, the system schematic diagram is shown in Figure 7.

#### 4.3.1. Vehicle Drive System

In any speed tracking control strategy, the reference speed plays an important role. The actual speed can perfectly track the reference speed provided that the reference speed is known ahead of time. Given the fact that the reference speed is related to the drive torque derived from the drive system, it is necessary to analyze this system. 

The drive system of a DDTV consists of driver operation devices such as the acceleration pedal and braking pedal, and dual-drive motors. The driver’s intention can be explained as the required speed or torque of the drive motor. Usually, a wheel-side reducer exists between the drive motor and drive wheel. Thus, the output drive torque of the drive system can be denoted as:

(18) $$T_{d}^{\left(l,r\right)}=T_{m}^{\left(l,r\right)}\cdot i_{wr}$$

where, $T_{m}^{\left(l,r\right)}$ represents the torque of the left and right side drive motor, respectively. $i_{wr}$ is the ratio of the wheel-side reducer. The output torque $T_{d}^{\left(l,r\right)}$ of the left and right side drive system are measured by two torque sensors, thus the reference speeds can be calculated by Equation (19):

(19) $$T_{d}^{\left(l,r\right)}-T_{R}^{\left(l,r\right)}=\bar{J_{eri}^{\left(l,r\right)}}\frac{dw_{r}^{\left(l,r\right)}}{dt}$$

(20) $$\left\{ \begin{array}{l} \bar{J_{eri}^{l}}=\delta^{l}\cdot \bar{J_{eri}^{total}}\\ \bar{J_{eri}^{r}}=\delta^{r}\cdot \bar{J_{eri}^{total}} \end{array}\right.$$

where, $T_{R}^{\left(l,r\right)}$ is the road resistance torque of the left and right side drive wheel. $\bar{J_{eri}^{l}}$, $\bar{J_{eri}^{r}}$ are respectively the ERI of the vehicle calculated at the left and right side drive wheel. $\delta^{l}$, $\delta^{r}$ are the ERI distribution coefficient. In this paper, we focus on the straight-line running, therefore, $\delta^{l}\;=\;\delta^{r}=0.5$. That is to say, the ERI is equally distributed to the two side drive wheels.

#### 4.3.2. Load Emulation System

As for the load emulation system, the dynamics can be represented as:

(21) $$T_{d}^{\left(l,r\right)}-T_{D}^{\left(l,r\right)}-T_{n}^{\left(l,r\right)}=(J_{gb}+J_{D}+J_{c})\frac{d\omega_{t}^{\left(l,r\right)}}{dt}+B\omega_{t}^{\left(l,r\right)}$$

Implementing Laplace transformation on Equation (21), the transfer function of the system can be expressed as:

(22) $$G_{t}(s)=\frac{\omega_{t}^{\left(l,r\right)}(s)}{T_{d}^{\left(l,r\right)}(s)-T_{D}^{\left(l,r\right)}(s)-T_{n}^{\left(l,r\right)}(s)}=\frac{1}{J_{t}s+B}$$

where, $J_{t}=J_{gb}+J_{D}+J_{c}$, $J_{gb}$ and $J_{D}$ are the rotational inertias of the gear box and dynamometer, respectively. $J_{c}$ is the total rotational inertia of the shaft attachments composed of the double-diaphragm couplings, shafts and torque sensors. *B* is the damping coefficient of the load emulation system. These parameters can be obtained from the product introduction or using system identification method [40].

The frictional resistance torque $T_{n}$ is a nonlinear factor in this system. But it has nearly no effect on the system dynamics given the fact that the friction torque is much smaller than the drive torque and load torque since the tested DDTV is of high inertia and high power. For simplicity, the nonlinear impact of this friction torque is neglected, and its value is assumed to be zero in this work.

Torque response of the dynamometer is assumed to be a first order plus time delay model:

(23) $$T_{D}^{\left(l,r\right)}=\frac{1}{\tau s+1}\overset{*}{T_{D}^{\left(l,r\right)}}$$

where, $T_{D}^{\left(l,r\right)}$ is the actual output torque of the left and right dynamometer, $\overset{*}{T_{D}^{\left(l,r\right)}}$ is the desired torque of the left and right dynamometer given by the controller. τ is a constant, denoting the torque response time of dynamometer.

### 4.4. Control Strategy and Simulink Model

**Figure 8:**
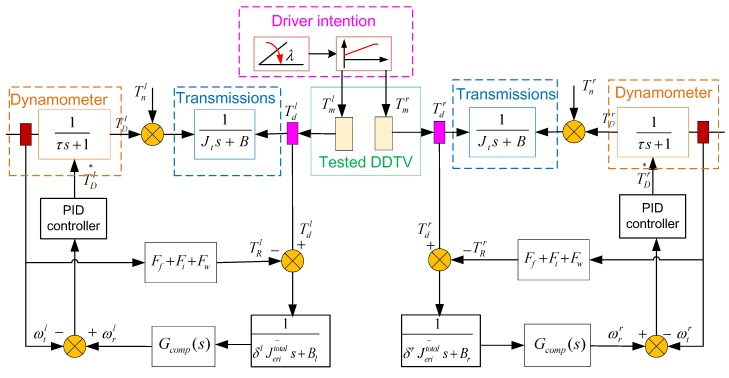
The structure of control strategy of the test bench.

**Figure 9:**
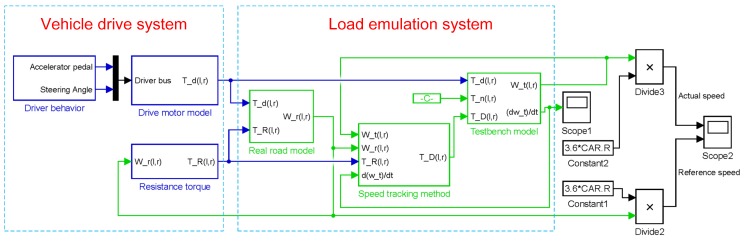
MATLAB/Simulink model of the single side test bench.

The structure of control strategy for the bench system is displayed in Figure 8. Its working principle can be described as follows: first, the two side drive torques $T_{d}^{\left(l,r\right)}$ and the test bench actual speeds $\omega_{t}^{\left(l,r\right)}$ are measured by torque sensors and speed sensors, respectively. The drive torques are subsequently used for the calculation of the reference speeds $w_{r}^{\left(l,r\right)}$. Next, the speed tracking controllers generate the target torque commands $\overset{*}{T_{D}^{\left(l,r\right)}}$ to the dual side dynamometers based on the difference of the current actual shaft speeds and reference speeds, to ensure the actual shaft speeds capable of tracking the reference speeds in real time. The speed tracking controller is a PID controller here. Figure 9 is the corresponding MATLAB/Simulink model of the single side test bench.

## 5. Experiment System Introduction.

### 5.1. General Setup

**Figure 10:**
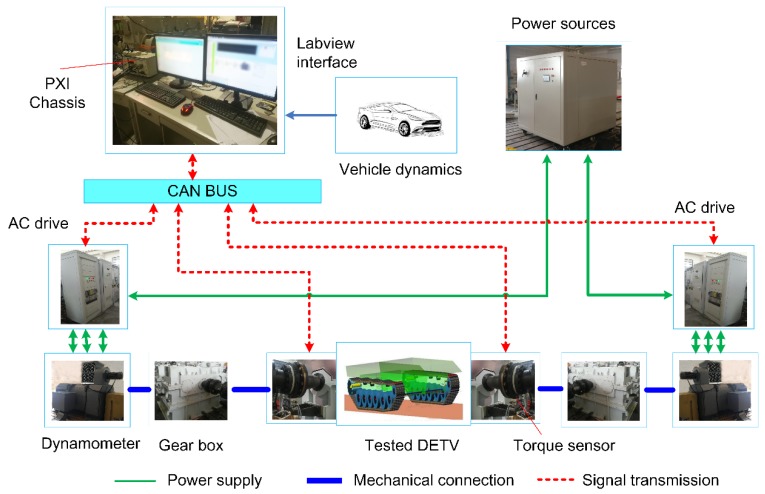
Block diagram of the test bench.

Figure 10 shows the designed test bench in this work. From a testing standpoint, the platform is mainly composed of two systems, the hardware system and the control system. The hardware system consists of two three-phase asynchronous squirrel-cage motors and their corresponding AC drives, two gearboxes and power resource. The asynchronous squirrel-cage motor with maximum power of 1200KW acts as the dynamometer to emulate the road load and the inertia road at the same time. The type of the AC drive is the ACS880 (ABB, Zurich, Switzerland). The control system mainly consists of a Siemens industrial personal computer (IPC, Berlin, Germany), a PXI chassis (National Instruments, Austin, TX, USA), two torque sensors (GIF, Alsdorf, Germany. 0~50,000 Nm, 0.1% FS) and other sensors. 

Real-time communication of the devices are realized by a controller area network bus (CAN bus). The test bench uses CAN protocol to integrate the IPC, double-side AC drives and sensors, ensuring the synchrony of the torque commands and read back the real-time torque and speed. A PXI chassis is used for real-time data acquisition. An IPC is employed for building and executing the vehicle model as well as implementing the control strategies. The test software is developed on the NI LabVIEW platform (version 15.0, National Instruments Inc., Austin, TX, USA). This software has plentiful functions such as real-time monitoring and displaying, as well as convenient data acquisition, processing and preservation.

### 5.2. Device Parameters

In this work, major parameters of the tested DDTV and the established test bench are listed in Table 1.

### 5.3. Test Contents

In order to validate the effectiveness of the proposed load emulation method, several experiments with different contents were carried out. The detailed testing groups are shown in Table 2, the test contents were designed for the following three aims. Firstly, in the first and the second group, the driving conditions were set the same while the vehicle mass were different, just for studying the robustness of the proposed method. Next, in the second to the third group, the vehicle mass was set the same while the driving conditions were different, just for studying the adaptability of the method to complex driving conditions. Finally, the test results from group 1 and group 2 are compared with the results obtained from simulation for verifying the accuracy of the established test bench Simulink model. 

## 6. Results and Discussion

### 6.1. Effectiveness Validation of the Dynamic Load Emulation Method

Under the same driving condition, if the total load torque of a vehicle on the test bench is similar to that of a vehicle on a real road within a minor error, the test bench could be considered a good performance in replicating the real road load conditions, thus the effectiveness of the load emulation method can be verified. According to Equation (3), the total load torque of a vehicle when running on a real road can be calculated as:

(24) $$\bar{T_{load}^{total}}=\bar{T_{R}}+\bar{J_{eri}^{total}}\frac{dw_{r}}{dt}$$

The load torques of the dual drive systems on a test bench are separately measured by two side torque sensors. So the total load torque of a vehicle on a test bench can be calculated as:

(25) $$T_{load}^{total}=T_{d}^{l}+T_{d}^{r}$$

where, $\bar{T_{load}^{total}}$, $T_{load}^{total}$ are respectively called the reference load torque and the actual load torque.

A parameter *μ* is defined as the relative error of the reference load torque and actual load torque. It is served as an indicator to evaluate the load emulation performance, and can be calculated as:

(26) $$\mu =\frac{\bar{T_{load}^{total}}-T_{load}^{total}}{\bar{T_{load}^{total}}}\times 100\%$$

#### 6.1.1. Straight Line with Different Vehicle Masses

**Figure 11:**
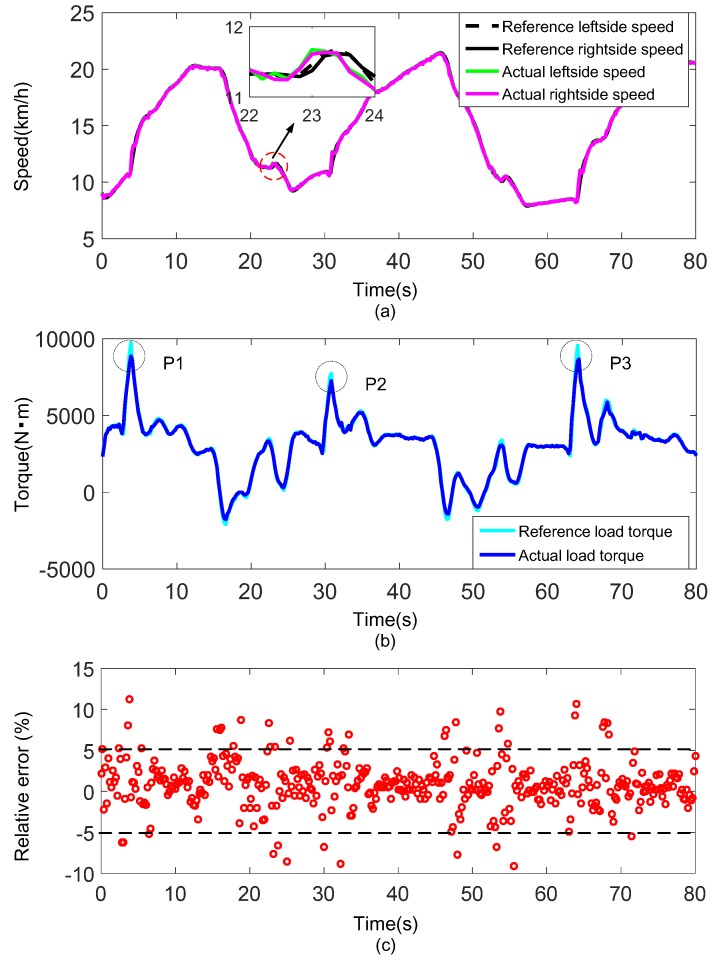
Experimental result under the driving condition of straight line with vehicle mass 20 t: (**a**) Speed tracking performance; (**b**) Comparison of total load torque of the vehicle; (**c**) Relative error of the load emulation (displayed by scatter plot).

**Figure 12:**
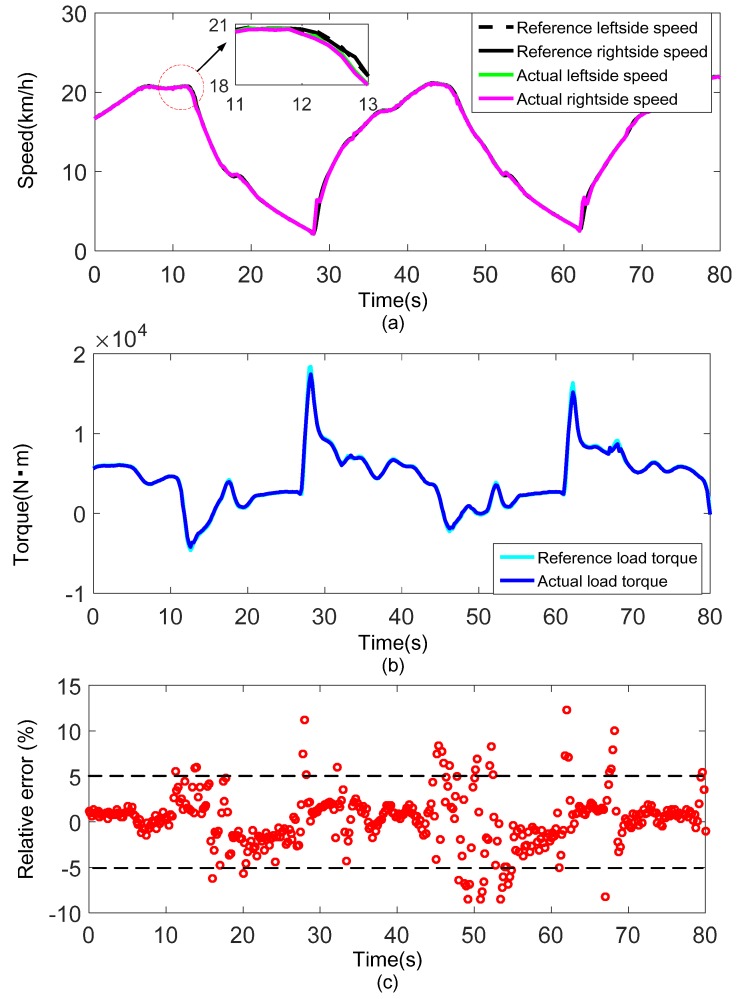
Experimental result under the driving condition of straight line with vehicle mass 30 t: (**a**) Speed tracking performance; (**b**) Comparison of total load torque of the vehicle; (**c**) Relative error of the load emulation (displayed by scatter plot).

The experimental results obtained under the test contents of group 1 and group 2 are shown in Figure 11 and Figure 12, respectively.

Figure 11a and Figure 12a show the reference speeds and actual speeds of both sides under the driving condition of straight line with different vehicle masses. The straight line is without slope, mild acceleration and deceleration intentions are executed alternatively on the vehicle. It is clear that the reference speeds are well followed by the actual speeds throughout the driving condition except for only few minor deviations. Additionally, from the partial enlargement of the figure, the actual speeds of both sides have a slight delay compared with the reference speeds. The speed-control loop delays, affected mainly by communication speed, control algorithm execution time and dynamometer response time, may account for this phenomenon. Figure 11b and Figure 12b show the reference load torque and actual load torque of the vehicle obtained by Equations (24) and (25). Generally, the actual load torque matches the reference load torque well. But it must be noted that the actual load torque is obviously lower than the reference load torque in region P1, P2 and P3 where sudden speed increases are observed in Figure 11a and Figure 12a. This is mainly a result of a poor response property of the dynamometer. The relative errors of the two torques throughout the driving condition are shown in Figure 11c and Figure 12c. Clearly, the relative errors are mostly concentrated within ±2%, almost all are within ±5%, indicating the good performance of the proposed load emulation method. Moreover, the experiments with different vehicle masses exhibit the similar good results demonstrates the robustness of the method.

#### 6.1.2. Straight Line with Slopes

**Figure 13:**
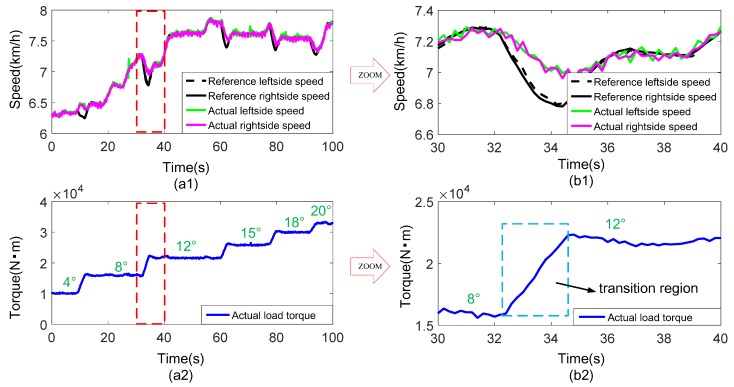
Experimental result under the driving condition of straight line with slopes with vehicle mass 30 t: (**a1**) Speed tracking performance; (**a2**) Actual total load torque of the vehicle on the test bench; (**b1**,**b2**) are partial enlargements of the figure (**a1**,**a2**), respectively.

In order to verify the adaptability of the proposed load emulation method to complex working conditions, a driving condition containing slopes is designed. The slope angle gradually increases from 4° to 20°, and the experimental result is shown in Figure 13.

According to Figure 13(a1), the actual speeds can generally track the reference speeds well throughout the whole driving condition. The straight line is with slope, and a slopping intention with the angle increasing from 4° to 20° is continuously implemented on the vehicle. Figure 13(b1,b2) are respectively the partial enlargements of Figure 13(a1,a2), which display the detailed test information when the slope angle varies from 8° to 12°. In these figures, it is obvious to see that the speed decreases while the load torque gradually increases at the instant transition stage of the two slope angles. This situation is similar to the one on a real road. However, the tracking performance is a little poor at this stage. The reason may be like this. The instantaneous change of the slope angle leads to the transient variation of the road resistance torque according to Equation (2), and the variation of this torque changes so fast that the dynamometer could not make an immediate response. This performance can be improved by applying a higher performance hardware system and using a more optimized control algorithm. 

Table 3 is the load emulation performance under different slope angles. For a certain angle, due to slight changes of speed when a vehicle is running on the slope, the inertia load can be very small and dynamic load mainly consists of the road load. In this way, 1 second time range is considered for calculating the average relative error for each angle. As can be seen from the table, the load emulation errors under each slope angle are less than 2%, indicating that the dynamic load simulation method presented in this paper also shows a good application performance under the driving condition of straight line with slopes.

Above all, the effectiveness of the proposed dynamic load emulation method is adequately validated under the driving condition of straight line in which the longitudinal dynamics of a DDTV is only taken into consideration. The lateral dynamics such as steering also plays an important role of DDTV drive performance [42,43]. A steering experiment should also be considered in future study to make the validation more adequate though this paper pay more attention to the method application in the load emulation system instead of the vehicle dynamics in the vehicle drive system.

### 6.2. Accuracy Validation of the Test. Bench Simulation Model.

**Figure 14:**
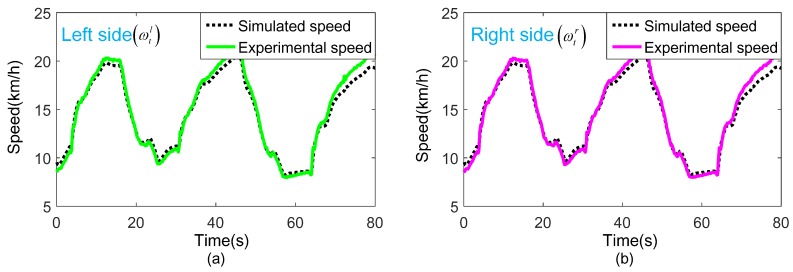
Comparison of the actual speed obtained by the group 1 experiment and the simulation model with vehicle mass 20 t: (**a**) Left side actual speed; (**b**) Right side actual speed.

**Figure 15:**
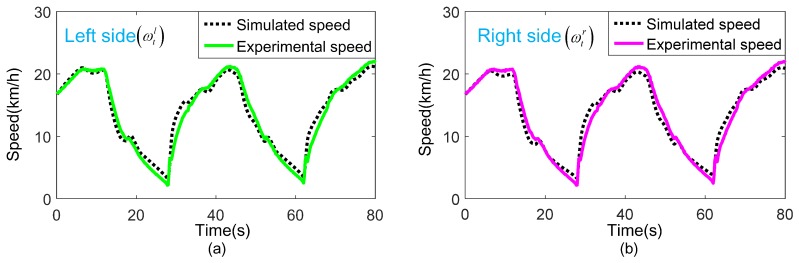
Comparison of the actual speed obtained by the group 2 experiment and the simulation model with vehicle mass 30 t: (**a**) Left side actual speed; (**b**) Right side actual speed.

Taking the actual torques ($T_{d}^{\left(l,r\right)}$) obtained from group 1 and group 2 experiments as the input of the test bench simulation model. The actual speeds ($\omega_{t}^{\left(l,r\right)}$) obtained by the simulation model and the experiment are compared as shown in Figure 14 and Figure 15.

Figure 14 and Figure 15 show that the speed obtained by the simulation model and the experiment matches very well in both sides with different vehicle masses, indicating that the simulation model is accurate. While, few minor deviations still exist. This is mainly because of the simplified operation in model establishment procedure, or inevitable external environment interference in a real bench test.

When designing a test bench for a DDTV, the simulation model can be used for beforehand research, such as the selection of dynamometer and gearbox, the optimization of control strategy, etc., thereby reducing the scheme changes and shortening the construction period.

## 7. Conclusions

This work innovatively focuses on the application of a dynamic load emulation method, denoted as speed tracking control strategy with feedforward compensation, on the design of a DDTV test bench featured with high power and large inertia. In this paper, the inertias of the vehicle body, tracks and roadwheels equivalent to the drive wheels are calculated separately to obtain an accurate vehicle total ERI. Dual dynamometers are applied to simulate the road load and the inertial load simultaneously. Experiments are conducted on the designed test bench under different test conditions. Results show that the proposed method has good robustness and adaptability to complex driving conditions, which demonstrates the effectiveness of the method application. 

Findings of this paper will contribute to potential research in related fields. The detailed described dynamic load emulation method can be considered as a flexible and accurate approach to be extensively applied to other test benches of dual-motor drive vehicles. Additionally, the MATLAB/Simulink model of the DDTV test bench is established based on detailed derivation of the system dynamics, which can be used for initial design of a test bench, thereby shortening the platform construction period. 

However, it must also be noted that at the instant of sudden transition in load torque, the speed tracking results exhibit slightly poor performance. This may be caused by the speed-control loop delays. Hence, a higher efficient hardware system and a more optimized control algorithm with consideration of nonlinear effect will be focused on in our future study. Moreover, lateral dynamics experiments such as steering, and real road test are needed to be conducted for further verifying the validity of the test bench response.

## Figures and Tables

**Table 1:** Major parameters of the tested DDTV and test bench.

Parameter	Value	Parameter	Value
Vehicle mass, *m*(t)	20, 30	Approach angle, $\varphi_{A}$	28.30
Drive wheel radius, *R*(m)	0.286	Departure angle, $\varphi_{D}$	30.10
Frontal area of the vehicle, *A*(m^2^)	4.84	Ratio of the wheel-side reducer, *i_wr_*	30
Aerodynamic drag, *C_D_*	1	Rolling resistance coefficient, *f*	0.05
Air density, *ρ*(Ns^2^/m^4^)	1.22	Rotational inertia of the dynamometer, *J_D_* (kg·m^2^)	42.60
Rotational inertia of the gear box, *J_gb_* (kg·m^2^)	491.10	Damping coefficient of the load emulation system, *B*(Nms/rad)	27
Total rotational inertia of the shaft attachments, *J_c_*(kg·m^2^)	7.95	Time constant of the dynamometer, *T*(s)	0.001
Maximum power of the test bench (kw)	2400	Sampling time the algorithm for dynamic load emulation (Hz)	500

**Table 2:** Test contents.

Group	Mass of the DDTV	Driving conditions
1	*m* = 20 t	Straight line without slope, *θ* = 0°
2	*m* = 30 t	Straight line without slope, *θ* = 0°
3	*m* = 30 t	Straight line with slope, *θ* = 4°, 8°, 12°, 15°, 18°, 20°

**Table 3:** Load emulation performance under the driving condition of straight line with different slope angles.

	Slope Angles					
	4°	8°	12°	15°	18°	20°
Time range (s)	3–4	20–21	48–49	71–72	85–86	97–98
Reference load torque (N·m)	10,173.59	16,163.96	21,587.55	25,693.44	30,045.67	33,130.49
Actual load torque (N·m)	10,034.42	16,018.54	21,215.22	25,188.42	29,829.72	32,680.25
Relative error	1.36%	0.90%	1.72%	1.96%	0.72%	1.35%
